# Health determining concepts important to people with Crohn's disease and their coverage by patient-reported outcomes of health and wellbeing^☆^^☆☆^

**DOI:** 10.1016/j.crohns.2012.12.014

**Published:** 2014-01-01

**Authors:** Mona Dür, Martina Sadloňová, Stefanie Haider, Alexa Binder, Michaela Stoffer, Michaela Coenen, Josef Smolen, Clemens Dejaco, Alexandra Kautzky-Willer, Veronika Fialka-Moser, Gabriele Moser, Tanja Alexandra Stamm

**Affiliations:** a Department of Internal Medicine III, Division of Rheumatology, Medical University of Vienna, Austria; b Institute for Medical Informatics, Biometry and Epidemiology, Ludwig-Maximilians-University, Munich, Germany; c Department of Internal Medicine III, Division of Gastroenterology and Hepatology, Medical University of Vienna, Austria; d Department of Internal Medicine III, Division of Diabetology, Medical University of Vienna, Austria; e Department of Physical Medicine and Rehabilitation, Medical University of Vienna, Austria

**Keywords:** DH, determinant of health, DHs, determinants of health, HP, health promotion, Crohn's disease, Determinants of health, Health and wellbeing, Health promotion, Inflammatory bowel disease, Patient-reported outcomes

## Abstract

**Background and aims:**

Busy clinical settings often restrict the possibility to focus on concepts that determine health in a positive way, commonly assessed by using patient-reported outcomes (PROs). We aimed to explore which determinants of health (DHs) are important to people with Crohn's disease (CD), to understand possible gender differences and to analyze whether these DHs are covered by PROs used in CD.

**Methods:**

Two systematic literature reviews were done to identify relevant DHs and clinically relevant PROs. We conducted a qualitative narrative biographical study and mapped the patients' experiences to concepts that determine health in a positive way. Experiences, DHs and the items of the PROs were compared by the WHO International Classification of Functioning, Disability and Health (ICF) as a common framework.

**Results:**

15 people with CD with a median age of 46 years (IQR 34–60) and median disease duration of 15 years (IQR 8–30) participated. Self-efficacy, social support, job satisfaction and occupational balance were mentioned most frequently. While participation appeared to have greater meaning to men, appreciation and resilience seemed to be more important for women. Of 18 PROs the *Perceived Stress Questionnaire* (PSQ), the *Inflammatory Bowel disease — Self*-*efficacy scale* (IBD-SES), the *Life Orientation Test — Revised* (LOT-R) and the Patient Activation Measure 13 (PAM-13) cover most DHs.

**Conclusions:**

This is the first study elaborating the coverage of patient's perspective by commonly used PROs in CD. The findings could support health professionals to focus on DHs in people with CD in clinical practice and research.

Summary box Significance of the study

What is already known about this subject?

• The positive effect of other determinants of health (DHs) on course of disease and health and wellbeing, such as of social support or coping strategies, is proven in people with Crohn's disease (CD).
• Patient reported outcomes (PROs) have been examined whether they cover patient's perspectives with other chronically autoimmune diseases.
• PROs have not been examined whether they cover patient's perspectives with CD based on qualitative data, nor if they cover DHs meaningful to people with CD, so far.

What are the new findings?

• Social support, self-efficacy, job satisfaction and occupational balance are DHs for people with CD.
• Social support is covered by five, self-efficacy by two PROs, *reflecting in an optimistic way*, is covered most often.
• Job satisfaction, occupational balance, secondary gain from illness, sense of coherence, vocational gratification, and work–life balance are not covered by any of the 18 identified PROs.
• The *Perceived Stress Questionnaire* (PSQ), the *Inflammatory Bowel Disease — Self Efficacy Scale* (IBD-SES), the Life Orientation Test — Revised (LOT-R) and the *Patient Activation Measure 13* (*PAM*-*13*) cover most DHs.

How might it impact on clinical practice in the foreseeable future?

• Other DHs beyond disease activity are important to people CD. Appreciation, coping, social participation, reflecting in optimistic way, resilience, self-efficacy, and social support can be assessed in clinical practice.
• Self-efficacy could be assessed routinely with the *IBD*-*SES* or the *PAM*-*13*, *social support is covered by the IBD*-*SES*, *the LOT*-*R and by the PSQ*.
• We recommend the use of the PSQ, the IBD-SES, the Lot-R and the PAM-13 in clinical routine to address HP in people with CD, to evaluate the need for HP interventions and their effect on the specific DHs.

## 1 Introduction

Crohn's disease (CD) is an inflammatory bowel disease (IBD) with a broad spectrum of clinical manifestations. CD may affect the entire gastrointestinal tract with discontinuous lesions involving all bowel layers. An irregular disease course with active and inactive periods is characteristic.^1^ CD may profoundly change or influence the patient's life situation, quality of life, health and wellbeing.^2^

Several determinants of health (DHs), such as social support and optimism are recognized to have an impact on the course and outcome of chronic inflammatory diseases.^3^, ^4^ DHs include the social, economic and physical environment, as well as the person's individual characteristics and behaviors, and can affect health either in a positive or in a negative way.^5^

There is a slight female predominance among patients with CD.^1^ In CD, nervous, endocrine and immune functions, the course of the disease, experiences and the consequences in the management of CD show differences between women and men.^6^, ^7^, ^8^

Health outcome research seeks to understand the end results of health care in a particular disease, an individual, or a group of patients. A scientific basis for understanding and studying health including patients' perspectives is needed.^9^, ^10^, ^11^ Patient reported outcomes (PROs) assess patients' perceptions within various dimensions of health, which still may deviate from patients' priorities for improvement of health.^12^ Other issues of health and well-being, such as DHs, beyond clinical or pathophysiologic aspects of a disease, may however be important to the people.^10^

Qualitative research allows gaining deeper understanding of the life world of other people. In the development and validation of PROs, this perspective is essential, otherwise PROs may lack issues important to patients that health professionals have not thought about. Qualitative research is needed to identify the concerns of people living with CD.^9^ Qualitative research differs fundamentally from quantitative research in that it generates knowledge about the individuals' experiences, attitudes, views and preferences, taking an in-depth approach to the issue it studies in order to understand it more thoroughly.^13^

The importance of DHs may change over time and disease course. Thus DHs need to be explored within a long-term perspective over the course of life. This allows understanding the current situation and experiences of an individual taking into account the whole life story. The narrative biographical method is a research method that combines the past experiences with the present life context.^8^, ^14^

Patients' perspectives have not been included into the development of CD-specific PROs so far. An examination whether they cover patients' perspectives based on qualitative data was not done yet.^15^, ^16^ Furthermore, potential gender differences in the experiences of living with CD have not been reported.

The research questions of this paper were, therefore: i) Which DHs are meaningful for people with CD and are there gender differences? ii) Are these DHs covered by PROs used in people with CD?

## 2 Materials and methods

### 2.1 Design

**Figure 1:**
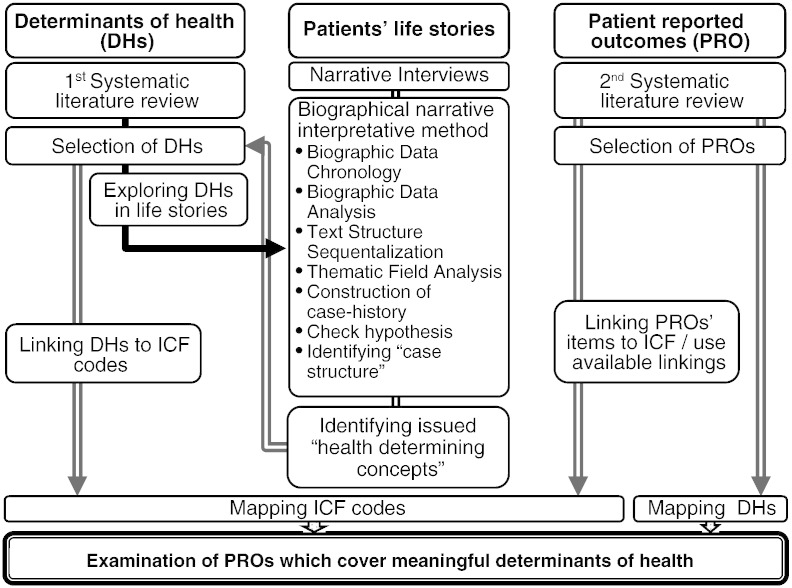
Design and method. Abbreviations: ICF = International Classification of Functioning, Disability and Health.

We conducted: (i) a first literature review to identify concepts that determine health in a positive way in patients with CD, (ii) a second literature review to determine PROs used in CD and (iii) a qualitative narrative biographic study to explore the experiences of people with CD in daily life over the course of their life time. DHs, the items of the PROs and experiences of people with CD were compared by using the WHO International Classification of Functioning, Disability and Health (ICF)^11^ as a common framework. Fig. 1 depicts a scheme of the method.

### 2.2 Literature review to select specific determinants of health

The first systematic literature review was done to identify concepts that determine health in a positive way. The inclusion criteria for a concept were: 1) mentioned in more than five publications, 2) valid or single and generally agreed definition available (such as from WHO), 3) evidence of its positive impact on health and 4) a relationship to functioning in daily life. In autumn, 2011 the databases PubMed, Web of Science, PsycINFO, and WHO IRIS were searched for the following keywords: DH, health promotion, and those DHs which were identified in a preliminary search to find appropriate search terms (such as social support, self-efficacy etc.).

Based on the selected list of DHs, we explored whether each DH was mentioned in the life stories of people with CD. This step was done by two researchers (MD, MS and/or TS) independently. In case of disagreement, each case was discussed in the research panel of three people who made a final informed decision. Frequencies of the concepts covered in the life stories were calculated using SPSS.^17^

### 2.3 Qualitative narrative analysis

#### 2.3.1 Participants

People with diagnoses of CD^18^ were selected from our gastroenterology outpatient clinic; ‘Maximum variation sampling’^19^ was followed considering gender, age, level of education, professional status and disease duration. Criteria for participation were (i) no history of psychiatric and/or neuro-motor disease unless medically well controlled, and (ii) German as their first language.

#### 2.3.2 Data collection

Participants were interviewed by one of two trained people (MS, MD), skilled and experienced in interviewing techniques. Participants received one general interview question asking them to tell their life stories and followed by specific questions regarding the told life events. Interviews lasted between one and 2.5 h. Participants were interviewed twice in order to give the interviewee and the interviewer the opportunity to reflect on what was said in the first interview session. All interviews were tape recorded and transcribed verbatim.

The transcripts were analyzed using the biographical narrative interpretative method which combines the exploration of the experiences of an individual from the perspective of the told narrative life-story and its relation to the biographic data.^20^ It is a conceptual analysis of the impact of CD on people's interpretation of their life's experience and biography. The steps of the qualitative method are depicted in the mid column in Fig. 1. Rigor and accuracy of this analysis were established by writing a reflective research diary by the first author and by using a research panel of at least three people to generate hypotheses regarding the meaning of what was told by the participants.

### 2.4 Literature review to identify PROs

The second systematic literature review was performed to identify PROs in CD. The following inclusion criteria for a PRO were applied: 1) assessing functioning, health and wellbeing, and/or one or more DH, 2) specifically developed for or validated in patients with CD, 3) published in a peer-reviewed journal and 4) published in English. In spring 2012, PubMed, CINAHL, PsycINFO were searched using the following keywords: CD, assessment, instrument, inventory, outcome measure, PRO, questionnaire, survey, scale and the specific DH (see Table 1). Descriptive, evaluative, and psychometric studies, reviews and articles that report the use of PROs in CD were selected. Case reports, economic evaluations and primary prevention studies targeted to so called healthy people (without a chronic disease) to prevent the development of IBD, and tools which considered disease activity or course of disease only were excluded.

**Table 1:** Definitions of the selected determinants of health.

Determinants of health	Definition
Appreciation	The construct of appreciation, the perception of popularity/acceptance by one's peers is typically used in the context of children and adolescent; it bases on feelings of social inclusion and of connectedness.^45^
Coping	Coping means constantly changing cognitive and behavioral efforts to manage specific external and/or internal demands that are experienced as stressful or exceeding the resources of the person.^46^
Vocational gratification	This term bases on the equity theory and the notion of contractual reciprocity. Vocational gratification can be defined as the experience of pleasing or satisfaction with the reward for the given effort in the context of employment.^47^
Secondary gain from illness	The term gain means the advantages of an illness experienced by the patient and that hinder recovery are generally termed “gain”. “Secondary gain from illness” defines a preconscious holding on to the illness because of supposed or real advantages.^48^
Job satisfaction	It is a pleasurable or positive emotional state resulting from the appraisal of one's job or job experience.^49^
Occupational balance	It is understood as a balance of social demands. Then an individual is experiencing (a) challenging and relaxing activities, (b) activities meaningful for the individual and activities meaningful in a sociocultural context and (c) activities intended to care for oneself and activities intended to care for others.^50^
Societal participation	Societal participation is the person's involvement in a life situation and in relations to other people (participation in society).^11^
Optimism	Optimism in the context of the current study means reflecting about one's life in a positive way despite a chronic disease respectively to have optimistic perspectives. It includes experiencing a chronic disease as a source for new challenges and the positive aspects – “benefits” – to having impairment.^51^, ^52^
Resilience	In psychology resilience refers to the idea of an individual's tendency to deal with stress and adversity. This may result in the individual “bouncing back” to a previous state of normal functioning, or using the experience of exposure to adversity to produce a “steeling effect” and function better than expected.^44^
Self-efficacy	It is our belief in our ability to succeed in certain situations. It is the individual's confidence in his or her ability to do a specific task or achieve certain outcomes.^53^
Sense of coherence	Sense of coherence means perceiving the world coherent. It consists of three components: comprehensibility, manageability and meaningfulness. Comprehensibility is a belief that things happen in an orderly and predictable fashion and a sense that you can understand events in your life and reasonably predict what will happen in the future. Manageability is one's belief that you have the skills or ability, the support, the help, or the resources necessary to take care of things, and that things are manageable and within your control. Meaningfulness covers a belief that things in life are interesting and a source of satisfaction, that things are really worth it.^54^
Social support	Social support refers to social connections, social network support and the frequency of social contact.^55^
Work–life balance	It is defined as the distribution of people's time between paid work and non-work activities — time with family, commuting, leisure and personal care.^56^

### 2.5 ICF-based analysis

**Figure 2:**
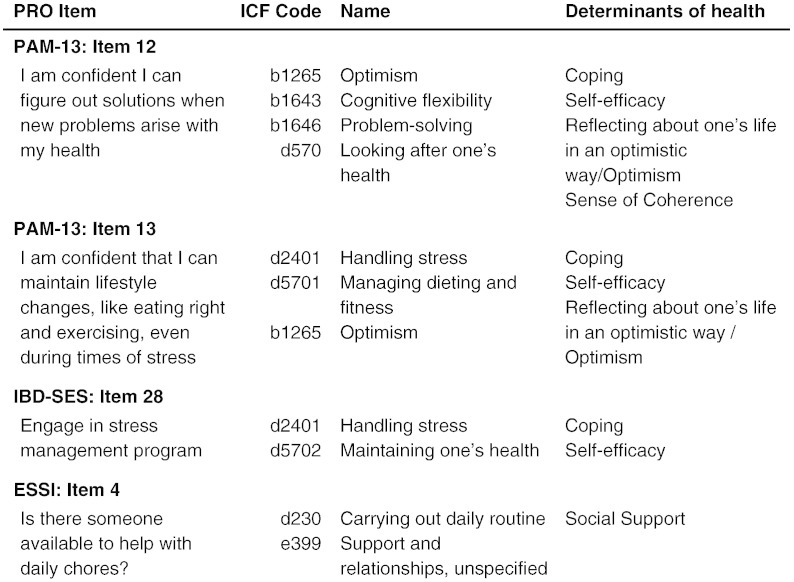
Examples for linking PROs' items to ICF codes and/or DHs. Abbreviations: ESSI = ENRICHD Social Support Instrument, IBD-SES = Inflammatory Bowel Disease — Self-Efficacy Scale, ICF = International Classification of Functioning, Disability and Health, PAM-13 = Patient Activation Measure 13 item version.

DHs and the extracted items of the PROs were compared based on the ICF. In the first step, the DHs as well as the items of the PROs were linked to the ICF categories by two researchers independently (MD, MC). The linking exercise followed a standard procedure and is similar to “translating” a concept or an item into “a common ICF language”.^21^ An example is given in Fig. 2. Due to the complexity of some of the selected DHs they were linked to as many ICF codes as necessary to describe them, such as self-efficacy given in Table 5, which is linked to four ICF codes. Additionally, available literature providing ICF codes linked to specific PROs was used.^15^, ^16^, ^22^, ^23^

### 2.6 Ethical considerations

Participants were informed about the study procedures and the ethical considerations. Written and oral informed consents were obtained. Confidentiality of the participants and the data was guaranteed. Therefore, the names given in relation to the quotes were changed. The study was approved by the ethic committee of the Medical University of Vienna, Austria.

## 3 Results

Based on the first literature search 13 DHs were selected, depicted and defined in Table 1.

In the qualitative study 15 people (eight women and seven men) with CD and a median disease duration of 15 months (IQR 8–30) participated. The median age was 46 years (IQR 34–60) and the median disease activity score assessed by the Harvey Bradshaw Index,^24^ was 7 (IQR 3–8), indicating a mild disease activity. Nine people were employed, five participants were retired, and one person was studying at the first interview. Demographical data of the participants per sex are depicted in Table 2.

**Table 2:** Demographic data of the participants per sex.

Age median (IQR) range	Disease duration median (IQR) range	Highest level of education	Professional status	Harvey Bradshaw Index^24^ (disease activity)
*Women (8)*				
43	9	L5	Employed	6 (mild)
38	22	L2	Employed	3 (remission)
34	22	L2	Employed	3 (remission)
27	6	L3	Employed	2 (remission)
59	29	L3	Retired	7 (mild)
63	1	L3	Retired	12 (moderate)
55	18	L3	Retired	7 (mild)
25	15	L2	Student	7 (mild)
40.5 (28.75–58) 25–63	16.5 (9–22) < 1–30			

*Men (7)*				
63	8	L3	Retired	15 (moderate)
37	4	L3	Employed	6 (mild)
25	9	L5	Employed	4 (remission)
46	30	L5	Employed	11 (moderate)
62	2	L3	Retired	1 (remission)
50	37	L3	Employed	23 (severe)
60	31	L3	Employed	6 (mild)
50 (37–62) 25–63	9 (4–31) 2–37			

L2 = secondary education first stage/second step of basic education, L3 = secondary education second stage/upper secondary education, L5 = first stage of tertiary education, not leading directly to an advanced research qualification,^57^ IQR = interquartile range.

The frequency of the DHs as well as gender differences found in the life stories are presented in a ranked order in Table 3. Both self-efficacy and social support were mentioned by 14 (93%), job satisfaction and occupational balance were both reported by 13 people (87%). These most frequently mentioned DHs applied to both sexes. While participation was of greater meaning for men compared with women (86 vs. 38%), resilience appeared to have more importance for women than men (63 vs. 29%).

**Table 3:** Frequency of determinants of health in the life stories in a ranked order.

DHs	Rank	*n*	f	m
Self-efficacy	1	14	7	7
Social support	1	14	8	6
Job satisfaction	2	13	7	6
Occupational balance	2	13	7	6
Participation (social)	3	9	3	6
Coping	4	8	4	4
Appreciation	4	8	5	3
Resilience	5	7	5	2
Reflecting in a positive way/optimism	5	7	4	3
Vocational gratification	5	7	3	4
Sense of coherence	6	6	3	3
Secondary gain from illness	7	3	1	2
Work–life balance	8	2	1	1
Total *n*		15	8	7

Abbr.: DHs: Determinants of health, f: female, m: male; *n*: number.

For example, Lukas described job satisfaction as:

“*It was fun*, *it was not*, *regarding satisfaction it was not gratifying completely*, *but the division and my colleagues were ok*. *Work was interesting always*, *salary was adequate*” (*Lines149*–*152*).

We identified occupational balance when Lukas talked about his meaningful activities:

“*I do a lot as handyman at home*, *with joy*, *not with a must*. *I start socializing*, *go to events and do gymnastics*. *What I am able to do*, *I do*” (*Lines 129*–*131*).

Lukas experienced a disease course which is typical for CD characterized by several flares, with occasional mild improvements of disease activity followed by recurrence of more active disease.^18^ In 2008, he experienced significant recrudescence, additionally he fell into a deep depression. A stay in a psychosomatic inpatient clinic for two weeks changed his life dramatically. He experienced self-efficacy regarding his health and wellbeing, as well as his disease course:

“*I had individual and group therapy*. *I*'*ve talked about my feelings*, *and about my pain and it* [*the pain*] *got better and better*, *until I had no*” (*Lines 101*–*105*). “*I felt better and better*, *no fatigue*, *regained strength*, *and started with sports again*, *I had become active again*” (*Lines 113*–*115*). “*In the past I have always been dissatisfied*, *because I could not make my own hours*, *work*, *or because I took on too much and I couldn*'*t manage it*, *then I was dissatisfied*. *Today*, *I do not plan in advance to do something*, *I just do it spontaneously*” (*Lines 160*–*162*).

After some years of living with the disease Lukas felt better than expected. We identified resilience in various sequences such as:

“*I was told that it will not get better*; *I will not recover*; *I thought there goes my whole life* (*Lines 167*–*170*). *Now*, *I am pleased with it*. *I got better and better*, *and currently I feel better than in all the years before everything had started* [*disease*]. *I am healthy*; *I am absolutely in best constitution*” (*Lines 178*–*185*).

Another example is given with Frank for whom social participation means to care for his health. His attendance at a Buddhist center was meaningful first and foremost to meet people, and participate and to be a part of a community:

“*We meet there at the weekend*, *the activities can be very exhausting*, *what*, *on the other hand*, *is real fun*, *I love to be there*. *I go there with a bored face*, *and leave happy*. *There are many people to exchange*, *I am glad that I have found that*, *it has become to a very important part of my life*” (*Lines 33*–*39*).

Sandra suffered from stomach-ache since her childhood, lost 16 kg of weight and was diagnosed with CD when she was 16 years old. Social support means a lot for Sandra and her disease management:

“*I could always lean on all of them* [*family*], *if there was something and I was unwell*. *Someone was there for my son*, *and my husband anyway*, *and the whole family actually*; *whenever I said I feel badly they were there*, *and they still are*” (*Lines 104*–*108*).

The linked ICF codes of the DHs are depicted in Table 5. An example of the linking process is given in Fig. 2. Five DHs, namely vocational gratification, job satisfaction, occupational balance, sense of coherence and work–life balance are *not covered* by the ICF.

In the second literature review 18 relevant PROs were identified and examined; they are listed and briefly described in Table 4.

**Table 4:** Characteristics of the identified patient-reported outcomes.

Abbr.	Patient-reported outcomes	Content	Items	Response options	Time frame
ADAPT	Assessment of the Demand for Additional Psychological Treatment^58^	Need for psychological treatment	12	Visual analog scales	Present
BDI-II	Beck Depression Inventory-II^25^	Depression	21	4 statements: increasing severity	Past, present, future
CPWDQ	Crohn's disease Perceived Work Disability Questionnaire^59^	Work capacity	14	4 statements: increasing frequency (1 = never, 4 all of the time)	Last year
DS-14	Type-D Scale^60^	Negative affectivity & social inhibition	14	Verifying statements (0 = false to 4 = true)	Present
EQ-5D	EuroQuoL Health questionnaire^26^	Health status	5	Statement (no, some, extreme problems) & visual analog scale	Present
ESSI	ENRICHD Social Support Instrument^61^	Extent of social Support	7	Question 1–6 (None, a little, some, most or all of the time), Question 7 (yes/no)	Present
FIQL	Fecal Inconsistency Quality of life Scale^62^	Health related Quality of Life	29	Different Likert scales	Present
HADS	Hospital Anxiety and Depression Scale^63^	Anxiety, depression	14	Frequency: 4-point Likert scale (0 = not at all, 4 = definitely)	Present
IBDQ-32	Inflammatory Bowel Disease Questionnaire^28^	Health related quality of life	32	7 point Likert scale (1 = significant impairment, 7 = no impairment)	2 weeks
IBD-SES	Inflammatory Bowel Disease Self-efficacy Scale^33^	Self-efficacy	29	10 point Likert scale (1 = not sure at all, 10 totally sure)	Present
LOT-R	Life Orientation Test-Revised^34^	Optimism	8	5 point Likert scale (0 = strongly disagree, 4 strongly agree)	Present
PAM-13	Patient-Activation Measure Short Form^64^	Health management skills, knowledge, confidence, motivation	13	5 point Likert scale (0 = strongly disagree, 4 strongly agree; 0 = poor, 4 = excellent)	Present
PSQ -V, -G	Perceived Stress Questionnaire^32^ recent form (PSQ-V)/general form (PSQ-G) covering the	Perceived stress	30	4-point scale on frequency (1 = almost never, 4 = usually)	Past month/past 2 years
PSQ-R	Perceived Stress Questionnaire Reconsidered^65^	Perceived stress	20	4-point scale on frequency (1 = almost never, 4 = usually)	Present
RFIPC	Rating Form of Inflammatory Bowel Disease Patient Concerns^66^	Worries, concerns regarding IBD	25	Visual analog scale (0 = Not at all, 100 = A great deal)	Present
SF-36	Short Form 36 Health survey^27^	Health related quality of life	36	Different response scales	4 weeks
SIBDQ	Short Inflammatory Bowel Disease Questionnaire^29^	Quality of life	10	7-point Likert scale on frequency (1 = all of the time, 7 = none of the time)	2 weeks
STAI	State-Trait Anxiety Inventory^67^	Anxiety about an event, and trait anxiety	40	Intensity 4-point Likert scale (1 = not at all, 4 very)	Present

The coverage of ICF codes for the Beck Depression Inventory (BDI),^25^ the EuroQuol Health Questionnaire (EQ-5D),^26^ the Short Form-36 Health Survey (SF-36),^27^ the IBD Questionnaire (IBDQ-32)^28^ and for the Short IBD Questionnaire (SIBDQ)^29^ were provided.^16^, ^22^, ^30^, ^31^ The examination of these PROs' coverage rests upon the quoted articles. The thirteen remaining PROs contained 324 items which were linked to 266 ICF codes; the mapping of the codes *personal factor* as well as *not covered* was not considered. None of the PROs covers all DHs, respectively linked ICF codes. The most frequent covered ICF code was b126 temperament and personality functions (10), followed by b1263 psychic stability (8). Social support is covered by five, self-efficacy by two PROs, reflection in an optimistic way, is covered most often. We recommend the use of the following four PROs due to the amount of covered DHs: the *Perceived Stress Questionnaire* (PSQ),^32^ the *Inflammatory Bowel Disease — Self efficacy scale* (IBD-SES),^33^ the *Patient Activation Measure 13* (PAM-13),^30^and the *Life Orientation Test — Revised* (LOT-R).^34^ Coping, reflecting in an optimistic way and self-efficacy could be assessed routinely with the PSQ, the IBD-SES and the PAM-13. Resilience is covered by the LOT-R, the PAM-13 and the PSQ. Social support is covered by the IBD-SES, the PSQ and the LOT-R. Moreover the LOT-R is the only PRO assessing social participation. The PSQ addresses most DHs (5). The coverage of the DHs by the PROs is shown in Table 5.

**Table 5:**
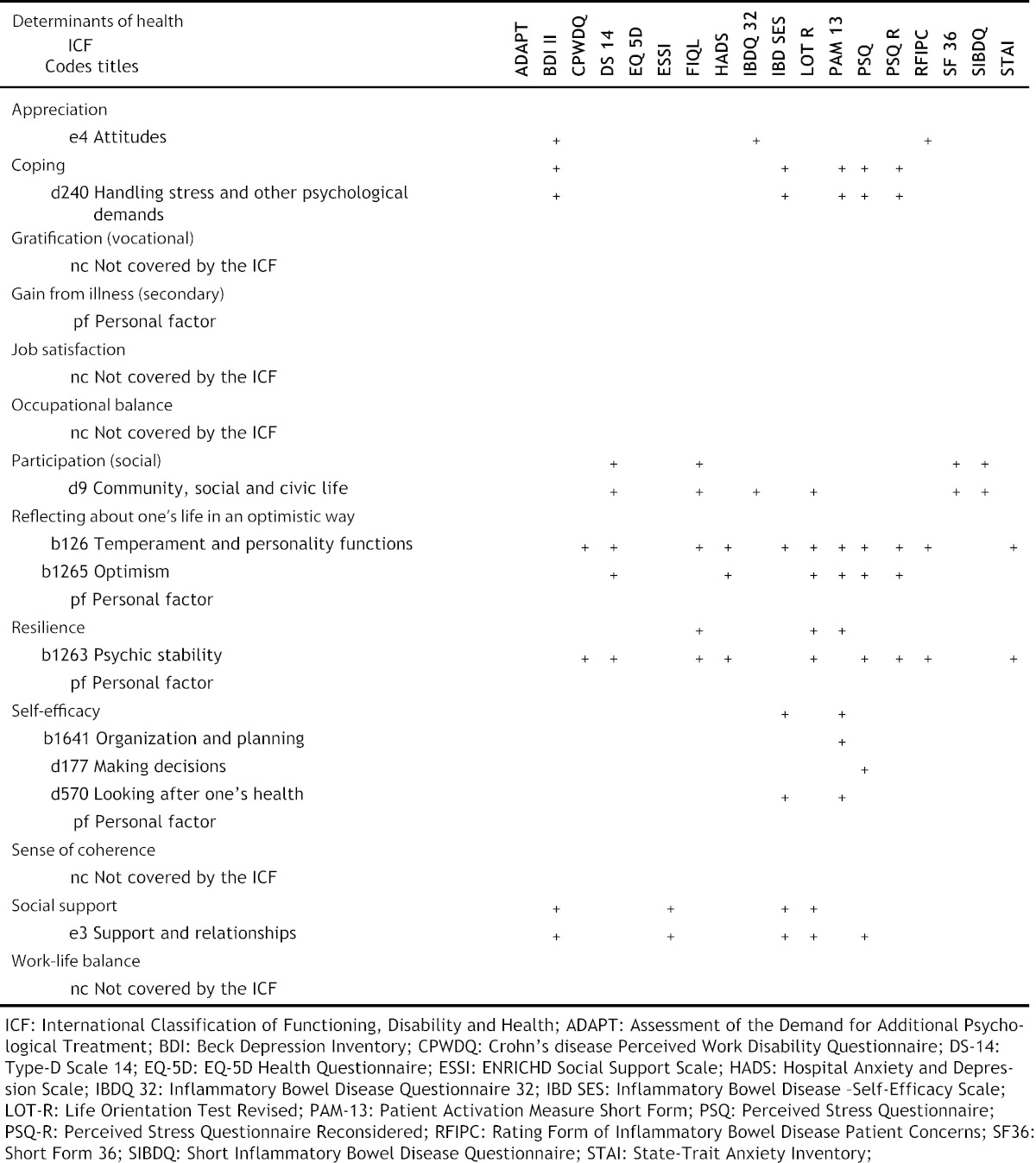
Coverage of the determinants of health by patient-reported outcome instruments.

## 4 Discussion

In this study we identified and critically appraised DHs which complement commonly used outcomes such as disease activity, from the perspective of people with CD. The examination of the 18 selected PROs applied in CD, showed that they cover few DHs which are meaningful for people with CD also.

It has been argued that outcomes in research and clinical practice should capture the perspective of patients with CD.^9^, ^10^ Our study explores the perspectives of patients with CD focusing on problems, but also on resources. PROs in CD have already been examined whether they cover several ICF codes.^16^, ^35^, ^36^ Their coverage of patient's perspective has not been explored as it was done in other chronic autoimmune diseases, so far.^37^, ^38^ To our knowledge, this is the first study examining PROs regarding patients' perspectives of people with CD based on qualitative research and regarding the coverage of DHs.

The latest NICE-guideline on CD focus on management and research in people with CD.^9^ However it does not give recommendations on which of the existing PROs should be used. Thus our paper suggests how to address other DHs beyond disease activity in CD. The use of the recommended PROs can support clinicians and health professionals to address other meaningful DHs.

Social support and self-efficacy should be considered to a greater extent in clinical practice and research, because in our study they were the most important DHs for the patients with CD. This is in accordance with literature as social support and self-efficacy were found to determine disease course and health.^33^, ^39^ In a related study in rheumatoid arthritis social support was important to 67% and self-efficacy 47%.^40^ Moreover, due to little evidence, other meaningful concepts such as job satisfaction and occupational balance require further research.^41^, ^42^, ^43^

The identified gender differences need further investigation and should be treated with caution due to small sample size. Gender differences of DHs in CD have not been reported elsewhere. In this study, women's disease duration was longer, even though they were younger than the men. Thus, women could have had more time to develop resilience. Men could have found it harder to maintain social participation, as found in other studies.^8^

The utilization of the PSQ, the IBD-SES, the LOT-R and the PAM-13 in clinical routine would be meaningful for patients with CD. The most suitable PRO appears to be the PSQ covering the most and meaningful DHs. The IBD-SES, the LOT-R or PSQ could be used to take account the meaning of social support (93%) and its substantial effect on health.^3^, ^44^ Self-efficacy that is also important to people with CD could be addressed by the use of the IBD-SES, PAM-13 and the PSQ.

### 4.1 Limitations

Qualitative research uses small sample sizes in order to generate meaning and to allow in-depth analyses of each case. However for generalizability of the findings quantification on larger samples in quantitative studies may be needed. Furthermore the use of other keywords and data bases could have led to different findings. A limitation might be that all participants were of one hospital.

When using the ICF as framework clarification is needed on how to deal with DHs which were a) partially covered, b) linked to personal factor, or c) not covered by the ICF. In this study we mapped these items according to the definitions of the DHs. Issues that are not covered by the ICF and are missing in the ICF should be included in the update process of the ICF which has already been initiated by the WHO. Furthermore there is a need for guidance of the process of mapping concepts which cannot be covered by the ICF, according to its purpose and definition.^21^

### 4.2 Implications of findings

This is the first study elaborating the coverage of patient's perspective by commonly used PROs in CD. Social support, self-efficacy and gender differences should get more attention in clinical practice and research. The use of the PSQ, the IBD-SES, the LOT-R or the PAM-13 is recommended.
